# Mapping the bacterial metabolic niche space

**DOI:** 10.1038/s41467-020-18695-z

**Published:** 2020-09-28

**Authors:** Ashkaan K. Fahimipour, Thilo Gross

**Affiliations:** 1 University of California Davis, Department of Computer Science, 1 Shields Ave, Davis, CA 95616 USA; 2 National Oceanic and Atmospheric Administration, Southwest Fisheries Science Center, 110 McAllister Way, Santa Cruz, CA 95060 USA; 3 Alfred-Wegener-Institut Helmholtz-Centre for Marine and Polar Research, AM Handelshafen 12, Bremerhaven, 27570 Germany; 4 Helmholtz Institute for Functional Marine Biodiversity (HIFMB), Ammerländer Heerstrasse 231, 26129 Oldenburg, Germany; 5 University of Oldenburg, Institute for Chemistry and Biology of the Marine Environment, Carl-von-Ossietzky Str. 9 - 11, 26129 Oldenburg, Germany

**Keywords:** Microbial ecology, Microbial communities, Community ecology, Ecological modelling

## Abstract

The rise in the availability of bacterial genomes defines a need for synthesis: abstracting from individual taxa, to see larger patterns of bacterial lifestyles across systems. A key concept for such synthesis in ecology is the niche, the set of capabilities that enables a population’s persistence and defines its impact on the environment. The set of possible niches forms the niche space, a conceptual space delineating ways in which persistence in a system is possible. Here we use manifold learning to map the space of metabolic networks representing thousands of bacterial genera. The results suggest a metabolic niche space comprising a collection of discrete clusters and branching manifolds, which constitute strategies spanning life in different habitats and hosts. We further demonstrate that communities from similar ecosystem types map to characteristic regions of this functional coordinate system, permitting coarse-graining of microbiomes in terms of ecological niches that may be filled.

## Abstract (web summary)

The ecological niche of a given microbe is difficult to define, but can be approximated from the range of biochemical reactions encoded by its genome. Here the authors use these genomic data and analyze them using manifold learning, which generates a diffusion map of the metabolic niche space of over 2500 bacteria.

## Introduction

It has been pointed out that a key to understanding the rules of life in ecological communities is to understand the structure of the niche space, the sets of ecological strategies that enable populations to grow and reproduce in an ecosystem^1–6^. Conceptual theories envision the niche space as an *n*-dimensional geometrical shape^1,7^ where each dimension is spanned by variables representing, often nonlinear combinations of salient traits or environmental features^8–11^. Empirical characterizations of the niche space have so far been conducted with a focus on individual groups of macrobiotic species, where different data analysis methods have been used to organize sets of functional traits that associate with major ecological roles in a system^11,12^; included are lizards^5^, beetles^13,14^, neotropical fish^6^, and terrestrial vascular plants^10,15^.

Bacteria are an attractive target for examining niche-based theories in ecology^16–20^ as many of the relevant traits, such as the ability to metabolize certain substrates or synthesize molecules that mediate ecological interactions, are biochemical in nature^21,22^. Hence they can be inferred from genomes, providing plentiful data to map the niche space on a grander scale. To operationalize the bacterial niche space we say that the sets of biochemical reactions encoded by genomes represent feasible metabolic strategies of extant microorganisms^5,23,24^. Together the strategies span a metabolic niche space^1^: the space of metabolic capabilities that populations may deploy to survive.

Ecological niches are thought to comprise complex nonlinear functions of multiple traits^5,10,11,25^. A central challenge in modeling the niche is thus to identify composite traits that map to interpretable ecological roles, or the ‘soft properties’^26^ that summarize organisms’ functional capabilities. A powerful analysis method for meeting this challenge is offered by diffusion maps^27,28^. This mathematically simple manifold learning method exploits the relationship between diffusion processes and geometric structures^29–31^ to define a new coordinate system for a dataset, where the axes, or variables, are nonlinear composites of its major features. The mathematical procedure does not provide an interpretation of these variables; however, our analyses show that they correspond to meaningful metabolic strategies. This offers a potential bridge between ecological niche theories and data that are readily accessible from bacterial genomes.

Here we use the diffusion map to construct and analyze a functional coordinate system that spans the bacterial metabolic niche space. As a compact prediction of metabolism, we generate genome-scale metabolic networks^22,32^ for representative species from all unique bacterial genera in the NCBI RefSeq^33^ release 92 database (*N* = 2621 genera). We map each representative network to a point in a 7769-dimensional discrete space, where axes indicate the presence or absence of predicted metabolic ‘traits’ given by unique chemical substrate–product pairs (i.e., directed edges) in the collection of networks. Although a complete picture of bacterial metabolism from genomic data is not yet possible, this array captures the major biochemical capabilities^34^ for a large fraction of known bacterial genera, and serves as input to the diffusion map algorithm. Our results indicate that manifold learning methods can delineate the salient geometric features^27,28,35^ of an ecological niche space, and that these structures mark potential strategies for survival under particular abiotic or biotic conditions. Subsequently, we demonstrate that bacterial communities from similar ecosystems occupy characteristic regions of the diffusion map, and that this provides a quantitative framework for defining potentially occupied ecological niches across complex microbial systems.

## Results

The diffusion map finds new variables that reflect nonlinear combinations of metabolic capabilities and returns them in the order of their importance (see Methods)^27,28,35^. Each variable assigns coordinate entries to the genomes that can then be used to order genera, from the most negative to the most positive entries, along curves that span the niche space. Dimensions in diffusion space can then be interpreted by analyzing the strategies of taxa near the extrema of the orderings^26,36^, corresponding to large positive or negative (i.e., far from zero) variable entries.

### Sharp differences delineate some metabolic strategies

**Fig. 1:**
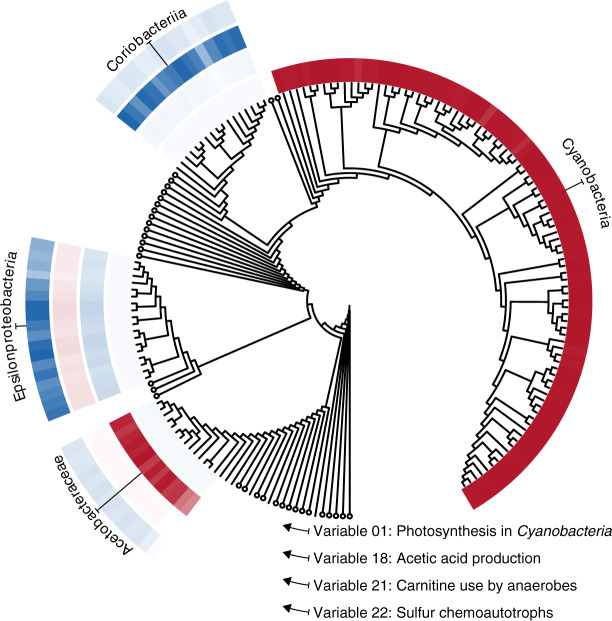
The diffusion map identifies variables describing discrete strategies. Variable entries for each genome are visualized as rings of colored tiles near the tips of a phylogenetic tree. Large negative or positive values (saturated reds and blues) indicate strong overlap with the focal strategy, whereas white indicates an absence of these capabilities. Circles are collapsed clades with near-zero entries in each of the four example variables. Clades receiving large negative or positive entries in any of the four example variables are expanded and annotated. The near-absence of semi-saturated tones indicates the strategies represented by these variables are approximately yes-or-no properties encoded by taxa.

The most important variable identified by the diffusion map, variable 1, separates the metabolic strategies of photosynthetic Cyanobacteria from those of all other taxa: the 108 cyanobacterial genomes in the dataset are assigned low values (i.e., negative numbers with large magnitudes) in variable 1, while all others have values that are close to zero (Fig. 1; Supplementary Fig. 1A). To confirm that this variable detects cyanobacterial photosynthetic activity, we identified metabolites that were over-represented in the metabolic networks of genera receiving far-from-zero entries in variable 1 (see Methods). This revealed an enrichment of 2-Phosphoglycolate, which is involved in essential photorespiratory pathways in photosynthetic organisms^37^; ribulose-1,5-bisphosphate (RuBP), used for carbon fixation from RuBisCO during photosynthesis; cyanophycin, a unique nitrogen reserve polymer^38^; and sucrose 6-phosphate, which catalyzes the final step in sucrose biosynthesis in Cyanobacteria^39^, confirming that the variable indicates the extent to which Cyanobacteria fix carbon through photosynthesis (Fig. 1; Supplementary Table 1).

The sharp differences in variable 1 show that this photosynthetic lifestyle is a discrete yes-or-no metabolic strategy where little middle ground exists. The diffusion map defines further variables that indicate such discrete clusters of unique capabilities (Fig. 1)—so-called ‘localized’ variables^40^—including capabilities associated with acetic acid production^41^ (variable 18), carnitine use for stress tolerance among anaerobic animal associates^42^ (variable 21), and chemolithoautotrophic or sulfur-oxidization strategies deployed by Epsilonproteobacteria near marine sediments and sea vents (variable 22).

### Contrasting the major strategies of host associates to life in soils and oceans

**Fig. 2:**
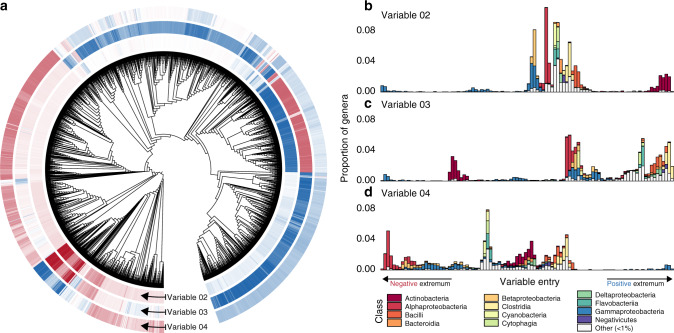
A spectrum of class-level capabilities indicated by variables 2, 3, and 4. **a** Variable entries for each genome are shown as rings of tiles near the tips of a phylogenetic tree. Darker red and blue tiles mark genomes receiving larger (in magnitude) negative and positive variable entries; white tiles mark near-zero entries. **b** The ordering of taxa defined by variable two entries, from negative to positive (left to right). The taxonomic compositions corresponding to variable entries are shown for each of 100 equally spaced bins. **c** The ordering of taxa defined by variable three entries. **d** The ordering of taxa defined by variable four entries. The variety of different values of these variables indicates a gradual shift in metabolic capabilities.

Some variables identified by the diffusion map analysis span a continuous spectrum of strategies, which align with major taxonomic classes. The most important of these are variables 2, 3, and 4, which contrast different putative metabolic strategies encoded by relatively large proportions of the analyzed genomes (Fig. 2; Supplementary Fig. S1B). For instance, variable 2 identifies major differences in predicted strategies among host-associated Gammaproteobacteria and soilborne Actinobacteria. Close relatives of pathogenic *Enterobacter*, *Franconibacter*, and *Buttiauxella* species^43^ score the lowest (i.e., most negative) values (Fig. 2a, b). Metabolic capabilities associated with these taxa include the synthesis of membrane phospholipid precursors common in Gammaproteobacteria like CDP-diacylglycerol^44^ and phosphatidylethanolamine, which may be involved in bacterial adhesion to host cells^45^; and the ability to metabolize uncommon sugars like L-lyxose^46^ (Supplementary Table 2). At the opposite end, we find primarily Gram-positive soil organisms from the *Microbacteriaceae*, *Beutenbergiaceae*, and *Micrococcaceae*^47^ (Fig. 2a, b). Among the most correlated capabilities for species near this extremum are the synthesis of decaprenyl diphosphate, a key component of cell wall biosynthesis in some taxa^48^; and compounds related to the synthesis of thiol and bimane derivatives, which can function as defenses against alkylating agents, oxygen stress, and antibiotics^49^ (Supplementary Table 3).

The Gammaproteobacteria genera that received the lowest entries in variable 2 also constituted the negative extremum of variable 3, and the positive extremum of variable 4 (compare Fig. 2a–d), suggesting that the bacterial metabolic niche space features a collection of low-dimensional manifolds that cross each other at branching points^36^. This branching point in particular illustrates a multiway contrast between a subset of the Gammaproteobacteria and at least 3 other taxonomic classes. At the positive end of variable 3, we find taxa representing mammal- and bird-associated Clostridia, Tissierellia, Erysipelotrichia, and Bacilli^47^. Characteristic metabolites of these genera include components of the Wood–Ljungdahl pathway^50^, enabling the use of hydrogen as an electron donor; and indole, a signaling molecule that has been shown to modulate host inflammation and interspecific competition in human gastrointestinal tracts^51^ (Supplementary Table 4). Our interpretation is that variable 3 identifies different potential strategies for colonizing and weathering stress or interspecific competition in animal hosts.

The species that score the lowest (i.e., most negative) values in variable 4 are epipelagic and marine Rhodobacterales, Rhizobiales, and Rhodospirillales that are capable of utilizing a broad spectrum of carbon sources^52^. Here the most significant metabolic reactions are all involved in the L-2-aminoadipate pathway of lysine synthesis^53^ and the production of L-pipecolic acid (Supplementary Table 5), pointing to a strategy for growth under high-salt conditions^54^. Our interpretation is that this variable traces a range of strategies spanning a generalist lifestyle in oceans to associations with terrestrial hosts.

Host-microbe interactions also feature in variables 8 and 10, which highlight endosymbionts and endoparasites with the smallest genomes in the dataset. The lowest values of variable 8 coincided with animal- and plant-associated Tenericutes^47^, as well as candidate genera like *Tremblaya* and *Sulcia*, that associate with insect bacteriocytes^55,56^. Among the top 10 markers of taxa scoring low values in variable 8 include the predicted uptake^22^ of key amino acids such as L-histidine, L-arginine, L-isoleucine, L-valine, L-lysine, and L-leucine (Supplementary Table 6). The negative extremum of variable 10 features obligate endoparastites and close relatives of opportunistic pathogens, including putative animal- and arthropod-associates of the *Pasteurellaceae*, *Erwiniaceae*, *Morganellaceae*, and *Rickettsiaceae*^47^. Similarly to variable 8, metabolic network features that distinguished this group include the predicted uptake of L-histidine, L-arginine, L-threonine, L-isoleucine, L-glutamine, and L-lysine (Supplementary Table 7). Together, these variables indicate that one widespread strategy for life in close association with animal or plant cells is the use of essential and non-essential host-derived amino acids^57^.

### Phylogenetic relatedness is a rough indicator of ecological similarity

The first several diffusion variables identify characteristic capabilities that discriminate between major taxonomic classes with many representative genera. To assess the overall relationship between metabolic similarity and phylogenetic relatedness we computed the correlation between pairwise inter-genome metabolic distances in diffusion space, and cophenetic distances on the phylogenetic tree (see ref. ^30^ for a detailed discussion of diffusion distances). Here a positive correlation suggests that closely related taxa deploy similar metabolic strategies on average.

**Fig. 3:**
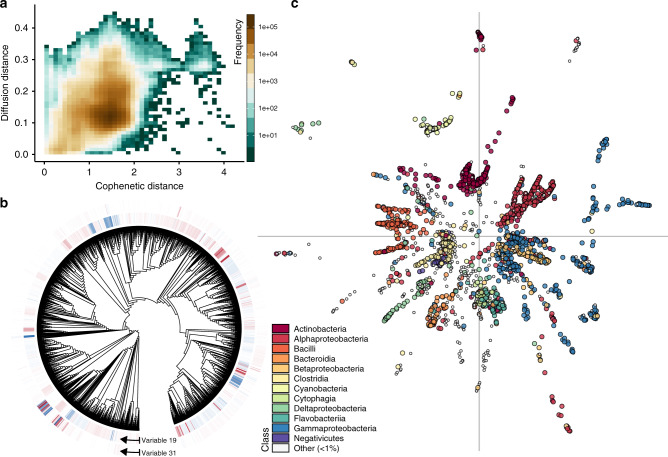
Metabolic and phylogenetic similarities are roughly correlated. **a** The correlation between distances in diffusion space and cophenetic distances between genome pairs (Mantel test, *r* = 0.273, *P* < 0.001). **b** Some variables such as 19 show similar functional capabilities shared by remotely related taxa (similar colors in distal parts of the tree). Others such as variable 31 highlight differences in closely related taxa, corresponding to the appearance of large positive and negative values (dark blue and red shades) in close proximity on the tree. **c** A 2-dimensional embedding of diffusion variables^36^, where individual genomes (points) are colored by taxonomic class. Axes mark (0, 0) in the coordinate system.

The Pearson correlation between distance matrices was positive but exhibited a small coefficient (Fig. 3a; Mantel test, *r* = 0.273, *P* < 0.001), marking a weak association between predicted metabolic capabilities and phylogenetic relatedness. While it is not surprising that phylogenies contain information on the ecological roles of microorganisms^58–60^, a visualization of this relationship highlights a caveat: a large range of diffusion distances are observed for most given cophenetic distances between genome pairs (Fig. 3a). This high degree of variance can be explained by the presence of diffusion variables that deviate from basic contrasts among major taxonomic groups (e.g., Fig. 2), including some that differentiate closely related taxa (Fig. 3b, Supplementary Fig. 1C), and those that show similar strategies among distantly-related taxa (Fig. 3b), potentially reflecting metabolic niche convergence^6^ or horizontal gene transfer.

These examples demonstrate that diffusion variables provide dozens or possibly hundreds of meaningful coordinates that trace the space of bacterial metabolic strategies. Using a procedure proposed by Moon et al.^36^ we combined diffusion variables in a low-dimensional visualization of the strategy space (Fig. 3c; Supplementary Fig. 1). This embedding recapitulates the result that phylogenetic relatedness offers only a coarse marker of predicted functional similarity, corresponding to the appearance of representatives from multiple classes in close proximity to one another in the niche space.

It is important to interpret lower-dimensional embeddings of high-dimensional data with caution^61^. However, multiple observations point to some consistent geometric structures in the bacterial metabolic niche space. Included are the results of a 2-dimensional embedding of diffusion variables (Fig. 3c; Supplementary Fig. 1), the presence of localized variables (e.g., Fig. 1) and crossing points (e.g., Fig. 2) in the diffusion map, and the results of enrichment analyses (Supplementary Tables 1–7). Namely, they point to a metabolic niche space consisting of multiple quasi one-dimensional branches rising from a common core, punctuated by discrete clusters of taxa with unique capabilities. This geometry may represent a conceptual hybrid between Hutchinson’s original idea of the niche space as a continuous hypervolume^1^, and modern ideas which postulate that sets of functional traits separate into discrete ecological clusters^5,6,12^. We conjecture that the putative filamentous structure has implications for our understanding of bacterial evolution and ecological functioning. For instance, the underlying branching geometry naturally leads to a large amount of unoccupied metabolic niche space (Fig. 3c). Similar gaps in niche space have been observed in macrobiotic communities^12^, and could correspond to bacteria that have yet to be sampled, isolated, or sequenced. Alternatively, they could be a result of ‘forbidden’ metabolisms, i.e., combinations of capabilities that may be suboptimal or even pointless for life in Earth’s ecosystems.

### Microbiomes map to characteristic regions of the metabolic niche space

Understanding the mapping from genomes to larger scale ecological strategies may prove useful for a variety of analyses^16–18^, such as quantifying the roles of organisms or designing substrates for culturing. Perhaps more importantly it provides an ecological frame of reference for coarse-graining complex bacterial communities. For a small scale demonstration of this point we created a simple mapping between a subset of community censuses from the Earth Microbiome Project (EMP)^62^ and our diffusion space.

**Fig. 4:**
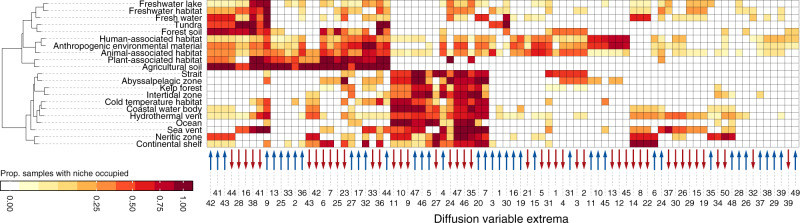
Bacterial communities map to characteristic regions of niche space. Metabolic niche profiles of samples from different ecosystem types (rows) in the Earth Microbiome Project^62^. Columns correspond to different diffusion variable extrema. Darker tiles indicate that a larger fraction of community censuses contained taxa that mapped to those extrema. Blue and red arrows along the horizontal axis denote positive and negative variable extrema respectively. A hierarchical cluster analysis groups ecosystems with similar niche profiles.

First, for each selected bacterial community census in the EMP we matched all taxa (16S rRNA gene amplicon sequence variants) to the most closely related genome considered by our diffusion map analysis, and retained matches that exhibited at least a 97% sequence similarity (see Methods). We then determined whether EMP communities contained at least one taxon that mapped to any of the 10 extremal genomes along any of the first 50 diffusion variables. As a result, each microbiome sample was characterized by the presence or absence of each of the first 100 extremal metabolic strategies. These presence-absence data represent ecological characterizations for individual EMP communities. To summarize further we computed the proportions of communities from different ecosystem types that displayed the different extremal strategies, resulting in a bacterial metabolic fingerprint for each ecosystem type (Fig. 4). These fingerprints can be used to study systematic differences in the functional capabilities of typical community members across habitats. For instance, a simple hierarchical clustering analysis of metabolic fingerprints groups different ecosystem types meaningfully together based on the metabolic strategies of their constituents (Fig. 4). Visible are clear strategy sets that differentiate functional diversity in freshwater, soil, marine, and host-associated systems.

## Discussion

Here we showed that the shape of a trait space can be systematized through manifold learning^27^. The diffusion map of bacterial capabilities reveals a wealth of ecologically salient variables that span a functional coordinate system. Some show evidence of discrete capabilities such as photosynthesis (Fig. 1). Other strategies span a continuous space representing degrees of specialization or reliance on hosts (Fig. 2). Yet others highlight strategies for energy production or stress response, some of which differentiate closely related species (Fig. 3b, Supplementary Fig. 1C) or emerged, potentially through convergent evolution or gene transfer, in different branches of the tree of life (Fig. 3b).

The diffusion variables provide a physical method for organizing the genomic information that continues to emerge, in a way that reveals both larger scale geometries and finer details compared to alternative embedding methods^27,36^ (Supplementary Discussion; Supplementary Figs. 1–6). From the perspective of microbial systems, diffusion distances in trait space (e.g., Fig. 3a) provide a powerful proxy for ecological similarities that can complement insights from current phylogenetic methods^60,63^. Traits used to calculate diffusion distances need not be derived from metabolic reconstructions of whole genomes as in the present analysis, but could comprise functional information identified, for instance, through species-level profiling^64^ of metagenomic or metatranscriptomic shotgun sequencing data. From an ecological point of view the present analysis constitutes the most extensive mapping of a niche space geometry so far, and facilitates the application of quantitative ecological theories to data describing bacterial communities.

Our analysis focused largely on the bacteria’s capabilities to catalyze steps in primary metabolism. Even within the realm of primary metabolism the genes reveal only the set of theoretical capabilities encoded by genomes, conceptually analogous to the fundamental niche concept^1^ in ecology. Hence our analysis ignores uncharacterized parts of secondary metabolism, behavior, regulation, and trophic interactions. For any other group of organisms such a limited analysis would be mostly meaningless; however, due to the diversity of metabolic capabilities in bacteria it reveals a rich and complex functional coordinate system (Fig. 3c). As our understanding of genomic data advances, deeper insights into secondary metabolism are bound to become available, providing an even more detailed picture of the metabolic niche space. Moreover, we envision that with future transcriptomic data, manifold learning methods could also map the realized niche^1^ (the metabolic strategies that are deployed under a given set of conditions) bringing our understanding of ecology in complex microbial communities closer to the biochemical level.

## Methods

### Metabolic networks

Genomes were obtained from the National Center for Biotechnology Information (NCBI) RefSeq^33^ release 92 database (accessed on 2019 March 20). We first obtained the ‘representative’, ‘reference’, ‘complete’, ‘contig’, and ‘scaffold’ sets and reduced these to a set of genus-level representatives using the following sampling procedure. We first selected a random representative genome for all unique genera in the combined ‘representative’ and ‘reference’ sets. Novel genera in the remaining RefSeq categories, that were not already represented in the ‘reference’ and ‘representative’ sets, were then appended to the set in the same way, for a total *N* = 2621 genomes. Metabolic models were constructed for the selected genome assemblies using the CarveMe reconstruction algorithm^32^, that starts with a universal bacterial metabolic model comprising known biochemical reactions in the BiGG Models^65^ database and generates genome-specific reaction sets by paring those without genomic support. Finally, metabolic models’ cytoplasmic compartments were retained and summarized as metabolic networks—directed graphs in which nodes are chemical metabolite compounds and directed edges link substrates to products^22^.

### Phylogenetic tree generation

Phylogenetic trees were used to visualize metabolic differences between taxa, and were constructed using the GToTree pipeline^66^ with the “universal” protein set defined by Hug et al.^67^. GToTree identifies target genes with HMMER3^68^, aligns them with MUSCLE^69^, and trims alignments with trimAl^70^. Trees were generated from the aligned and concatenated gene sets using FastTree^71^, and visualized using iToL^72^.

### Diffusion map procedure

Diffusion mapping^27,28^ was performed using the algorithm described by Barter & Gross^26^. Briefly, the method involves (i) calculating a matrix describing euclidean similarities among the *k*-nearest neighbors for samples in a dataset, (ii) interpreting this as a weighted adjacency matrix, and (iii) computing the corresponding row-normalized Laplacian matrix. The eigenvectors of the Laplacian represent new diffusion variables describing important variation in the dataset^26^. The importance of each eigenvector is indicated by the corresponding Laplacian eigenvalue^27,30^, which captures the characteristic time scale of diffusive modes over the data in that dimension^35^. The first (i.e., most important) variable is given by the eigenvector corresponding to the smallest non-zero eigenvalue, then the second smallest eigenvalue, and so on. This variant is nearly parameter-free, with only a single choice for the value of *k*. Here, we consider *k* = 10, although the results presented above were insensitive to the choice of *k*.

### Identifying associated metabolites

We sought to identify metabolites that were over-represented in the metabolic networks of taxa, that were themselves assigned extreme entries along diffusion map variables. This was accomplished using a permutational variant of the gene set enrichment analysis, GSEA^73^. Genome rankings were defined by the orderings specified by each diffusion variable. Enrichment analyses were accomplished for the ranked sets using the fgsea library in R^74^, with a Benjamini–Hochberg–adjusted^75^ *P* value < 0.05 used as the threshold for retaining metabolites associated with taxa that map to variable extrema.

### Mapping environmental samples to diffusion space

We obtained the ‘emp_deblur_150bp.subset_2k.rare_5000’ dataset, describing a subset of the environmental 16S rRNA gene sequences from the Earth Microbiome Project^62^, EMP, accessed via ftp://ftp.microbio.me/emp/. Communities from the EMP were mapped to diffusion space using the following procedure: First, we generated a BLAST^76^ reference database of predicted 16S rRNA gene sequences for our set of RefSeq genomes using barrnap (https://github.com/tseemann/barrnap) to identify and retain the first instance of this ribosomal gene. The DECIPHER library^77^ in R was used to align sequences. We then conducted a BLAST sequence similarity search to match denoised sequence variants present in each EMP sample to the custom BLAST database and retained the top hits. Niches—operationally defined as the strategies describing the 10 taxa with the highest (positive) and lowest (negative) entries along each diffusion variable—were said to be occupied by taxa in an EMP community census if at least one detected sequence variant exhibited a 97% or greater rRNA gene sequence similarity to any of the extremal genomes. The results of this procedure were summarized as plots of the proportion of samples within each EMP ‘env_feature’ category satisfying this criterion. Hierarchical clustering of similar ecosystem types was accomplished using the Ward^78^ linkage method.

### Reporting summary

Further information on research design is available in the Nature Research Reporting Summary linked to this article.

## Supplementary information

Supplementary Information

Reporting Summary

## Data Availability

Genome accession numbers are available at 10.6084/m9.figshare.12864011.v4.

## Code availability

R scripts and sample data are available at 10.6084/m9.figshare.12864011.v4.
