# Supplementary material for: Mapping the bacterial metabolic niche space
Source: Nat Commun. 2020 Sep 28;11:4887. doi: 10.1038/s41467-020-18695-z (PMC7522980; doi:10.1038/s41467-020-18695-z)
Supplement: Supplementary file 1 — Supplementary Information [file 41467_2020_18695_MOESM1_ESM.pdf]

Supplementary Information: Mapping the bacterial metabolic niche space

Ashkaan K. Fahimipour[1,2,*] and Thilo Gross[1,3,4,5]

[1] *University of California Davis, Department of Computer Science, 1 Shields Ave, Davis, CA 95616, USA*
[2] *National Oceanic and Atmospheric Administration, Southwest Fisheries Science Center, 110 McAllister Way, Santa Cruz, CA 95060, USA*
[3] *Alfred-Wegener-Institut Helmholtz-Zentrum für Polar und Meeresforschung, Oldenburg, DE*
[4] *Helmholtz Institute for Functional Marine Biodiversity at the University of Oldenburg, Ammerländer Heerstrasse 231, 26129 Oldenburg, DE*
[5] *University of Oldenburg, Institute for Chemistry and Biology of the Marine Environment, Ammerländer Heerstrasse 9 - 11, 26129 Oldenburg, DE*

Supplementary Discussion

Commonly used dimensionality reduction methods in biology include principal component analysis (PCA), multidimensional scaling (MDS), and t-distributed stochastic neighbor embedding (t-SNE) [1]. These methods provide valuable insights across a variety of applications (see ref. [2] for a comparison of embedding methods with high-throughput sequencing data). Here we present examples of their limitations in the context of metabolic trait space reconstruction. Consistent with prior results [2], we observe that PCA identifies some important global contrasts but misses fine details, that neighbor embedding methods like t-SNE find some localized [3] clusters by preserving local data structures but cannot reliably identify global structures, and that MDS is sensitive to noise.

Desirable properties of an ecological coordinate system

An effective ecological coordinate system for high-dimensional trait data would ideally preserve important local and global features of the dataset [2]. We demonstrate by way of example that diffusion-based methods learn both local and global structures in the trait space, while popular methods usually emphasize only one of these desirable properties.

We consider three examples of types of functional 'soft properties' [4] identified by the diffusion map and supported by enrichment analysis [5] (Supplementary Tables 1-7): an example of capabilities that uniquely distinguish a group from all others (variable

*To whom correspondence should be addressed: ashkaan.fahimipour@noaa.gov

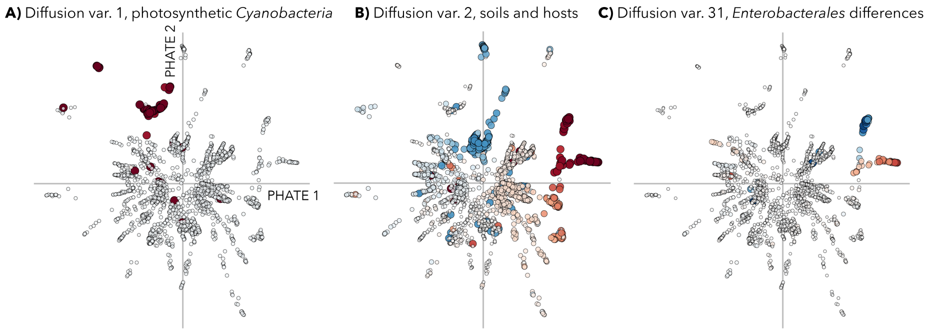

Figure 1: **Two-dimensional embedding of diffusion variables, computed using the 'PHATE' algorithm** [2]. Points are individual genomes colored by their assigned entries in a specific diffusion variable. Dark shades of red and blue correspond to small (i.e., the most negative) and large (positive) variable entries; white points are near zero. Axes mark (0, 0) in the coordinate system. **A)** Diffusion variable 1, identifying photosynthetic capabilities in Cyanobacteria (dark red points). **B)** Diffusion variable 2, which identifies differences between soil-associated Actinobacteria (dark blue) and host-associated Gammaproteobacteria (dark red). **C)** Diffusion variable 31, which finds a functional split among close relatives in the Enterobacterales.

1; carbon fixation by photosynthetic Cyanobacteria), an example of conserved differences between major taxonomic classes (variable 2; soilborne Actinobacteria vs. host-associated Gammaproteobacteria), and an example of major differences among close relatives (variable 31; differences among species in the Enterobacterales).

Supplementary Figure 1 shows the two-dimensional embedding of diffusion variables using the 'potential of heat-diffusion for affinity-based transition embedding' (PHATE) method (also see Fig. 3C in the main text) of Moon et al. [2]. Coloring the points (genomes) by diffusion variable entries shows how the method captures both intricate (Supplementary Figs. 1A, 1C) and global scale (Supplementary Fig. 1B) structures in the metabolic trait data in only two dimensions.

In contrast, other methods discard important fine-grained details encoded in higher dimensions. This is well-understood for linear methods like PCA that focus on explain-

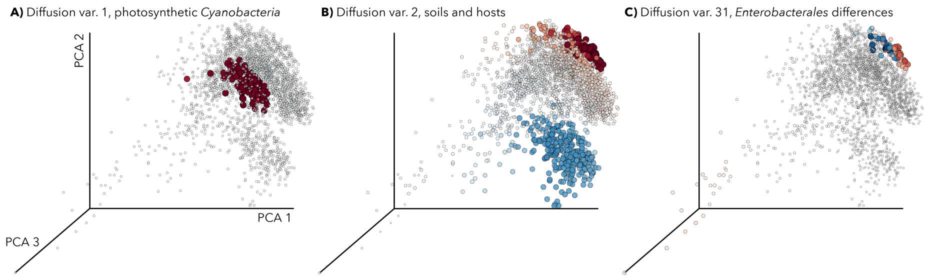

Figure 2: **Three-dimensional embedding of data points using principal component analysis.** Points are individual genomes colored by their assigned entries in a specific diffusion variable (see Supplementary Fig. 1 for a description). **A)** Diffusion variable 1, **B)** diffusion variable 2, and **C)** diffusion variable 31.

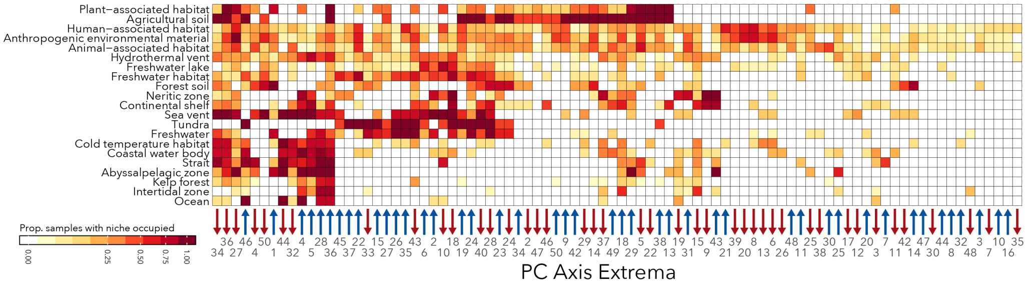

Figure 3: **PCA only reveals rough structures in trait data space.** Rows denote different ecosystem types in the Earth Microbiome Project dataset [6]. Columns correspond to different principal component axis extrema for the first 50 axes. Darker tiles indicate that a larger fraction of community censuses contained taxa that mapped to those extrema. Blue and red arrows along the horizontal axis denote positive and negative variable extrema respectively. Columns and rows are ordered based on the procedure described in *Methods*.

ing global variances [2] (Supplementary Figs. 2A-2C). In practice, this means that PCA is able to find some global contrasts in the trait dataset, like the major differences in metabolic capabilities between Actinobacteria and Gammaproteobacteria (Supplementary Fig. 2B). However, the method is unable to identify finer structures near nonlinear submanifolds. As a consequence, details like intra-class differences in metabolic traits are obfuscated (e.g., Supplementary Fig. 2C). This observation is recapitulated by a mapping between samples in the Earth Microbiome Project (EMP) [6] and PCA axes (see Fig. 4; description in Methods), which provides only a rough characterization of ecosystem types (Supplementary Fig. 3).

The t-SNE [1] method is capable of identifying localized [3] clusters in the data (e.g., differences in the Cyanobacteria; Supplementary Fig. 4A) but cannot reliably capture global features of the dataset. This is because t-SNE minimizes an objective function that ignores large dissimilarities between data points, causing distances in t-SNE space to be mostly meaningless [2]. This leads to issues particularly in the analysis of data that

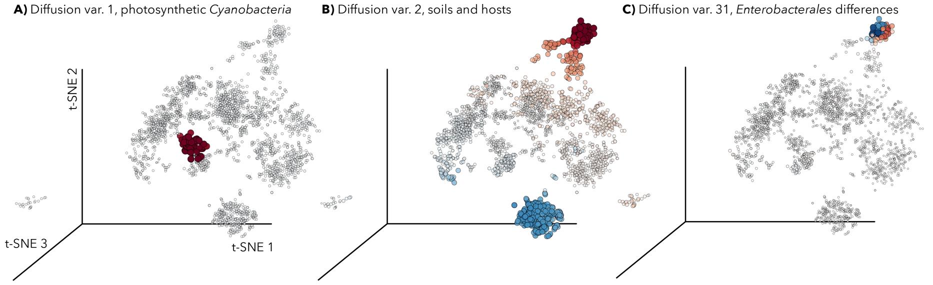

Figure 4: **Three-dimensional embedding of data points using t-distributed stochastic neighbor embedding.** Points are individual genomes colored by their assigned entries in a specific diffusion variable (see Supplementary Fig. 1 for a description). **A)** Diffusion variable 1, **B)** diffusion variable 2, and **C)** diffusion variable 31.

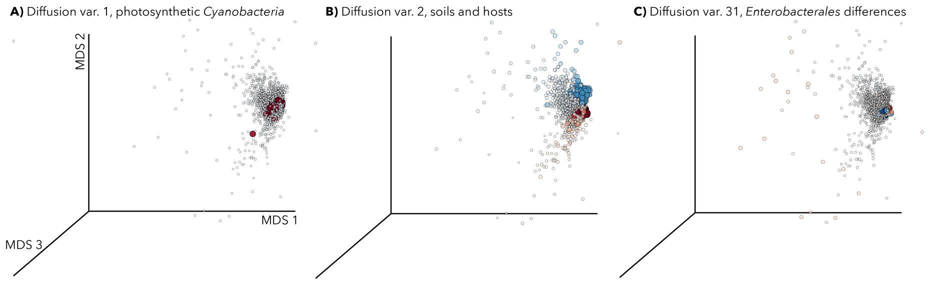

Figure 5: **Three-dimensional embedding of data points using multidimensional scaling.** Points are individual genomes colored by their assigned entries in a specific diffusion variable (see Supplementary Fig. 1 for a description). **A)** Diffusion variable 1, **B)** diffusion variable 2, and **C)** diffusion variable 31.

are well-described by continuous or branching trajectories (e.g., cell differentiation data), as t-SNE shatters these trajectories leading to the false impression of data clusters [2].

Robustness of diffusion mapping to data perturbations

A notable concern is the sensitivity of dimensionality reduction methods to noise [2]. This problem is particularly apparent in the application of multidimensional scaling to our metabolic trait dataset. Visible in the embedding is a strong sensitivity to outliers (Supplementary Figs. 5A-5C), leading to a tight cluster of genomes with very little visible structure. Within this cluster, important features are generally difficult to resolve.

In contrast, a majority of local structures in the diffusion map are robust to data perturbations. To demonstrate this point, we computed the degree to which replacing a specified proportion of real trait sets with random metabolic configurations altered the

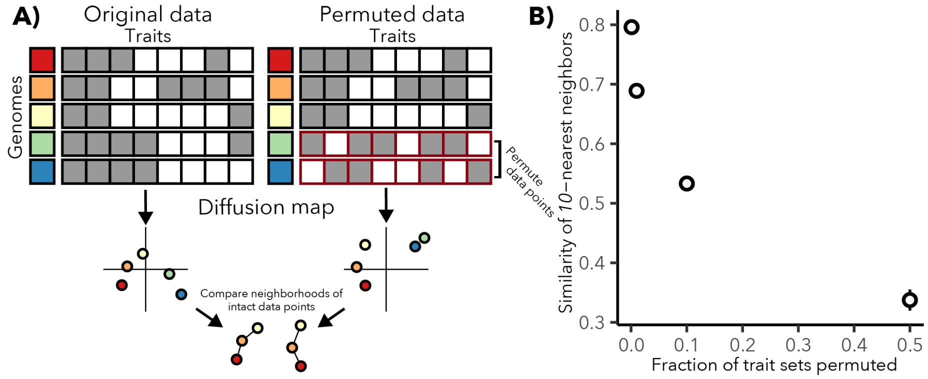

Figure 6: **Sensitivity of local structures in the diffusion map to data perturbations.** **A)** Schematic of the procedure. **B)** Results of the permutational analysis. The vertical axis shows the mean similarity of the 10−nearest neighbors for intact data points over 100 permutations (±2 SEM), measured as 1−Jaccard distance between pairs of neighbor sets [7].

neighborhoods of intact data points in diffusion space (see Supplementary Fig. 6A). Similarities of the 10-nearest neighbors between pairs of matching points in the intact and permuted diffusion spaces were calculated as $1 - D_J$, where D_J is the binary Jaccard distance [7]. Supplementary Fig. 6B shows the results of 100 iterations of the procedure outlined in Fig. 5A, which indicate that local structures are mostly preserved for the intact data (similarity > 0.5), even when permuting 10% of the trait data points. Thus, geometric structures in the diffusion map have the benefit of being robust to potential noise introduced by sequencing errors, genome and annotation incompleteness, or the metabolic reconstruction process.

Even using a limited number of examples, several of the limitations of common methods for trait space reconstruction become apparent. Included are tradeoffs between the preservation of local and global data structures, prior assumptions about the linearity or geometry of the underlying data, and sensitivity to noise.

Supplementary References

[1] Laurens van der Maaten and Geoffrey Hinton. Visualizing data using t-sne. *Journal of machine learning research*, 9(Nov):2579–2605, 2008.

[2] Kevin R Moon, David van Dijk, Zheng Wang, Scott Gigante, Daniel B Burkhardt, William S Chen, Kristina Yim, Antonia van den Elzen, Matthew J Hirn, Ronald R Coifman, et al. Visualizing structure and transitions in high-dimensional biological data. *Nature Biotechnology*, 37(12):1482–1492, 2019.

[3] Amy Nyberg, Thilo Gross, and Kevin E Bassler. Mesoscopic structures and the laplacian spectra of random geometric graphs. *Journal of Complex Networks*, 3(4):543–551, 2015.

[4] Edmund Barter and Thilo Gross. Manifold cities: social variables of urban areas in the uk. *Proceedings of the Royal Society A*, 475(2221):20180615, 2019.

[5] Aravind Subramanian, Pablo Tamayo, Vamsi K Mootha, Sayan Mukherjee, Benjamin L Ebert, Michael A Gillette, Amanda Paulovich, Scott L Pomeroy, Todd R Golub, Eric S Lander, et al. Gene set enrichment analysis: a knowledge-based approach for interpreting genome-wide expression profiles. *Proceedings of the National Academy of Sciences*, 102(43):15545–15550, 2005.

[6] Luke R Thompson, Jon G Sanders, Daniel McDonald, Amnon Amir, Joshua Ladau, Kenneth J Locey, Robert J Prill, Anupriya Tripathi, Sean M Gibbons, Gail Ackermann, et al. A communal catalogue reveals earth's multiscale microbial diversity. *Nature*, 551(7681):457, 2017.

[7] Shamus M Cooley, Timothy Hamilton, Eric J Deeds, and J Christian J Ray. A novel metric reveals previously unrecognized distortion in dimensionality reduction of scrna-seq data. *BioRxiv*, page 689851, 2019.

[8] Yoav Benjamini and Yosef Hochberg. Controlling the false discovery rate: a practical and powerful approach to multiple testing. *Journal of the Royal statistical society: series B (Methodological)*, 57(1):289–300, 1995.

[9] Elhanan Borenstein, Martin Kupiec, Marcus W Feldman, and Eytan Ruppin. Large-scale reconstruction and phylogenetic analysis of metabolic environments. *Proceedings of the National Academy of Sciences*, 105(38):14482–14487, 2008.

Supplementary Tables

Metabolite	Synthesized	Enrich. Score	FDR-Adj. P
2-Phosphoglycolate	Yes	−1.073	0.0013
D-Ribulose 1,5-bisphosphate	Yes	−1.071	0.0013
Cyanophycin	Yes	−1.070	0.0013
1,5-Diaminopentane	Yes	−1.067	0.0013
Sucrose 6-phosphate	Yes	−1.067	0.0013

Table 1: Top 5 over-represented metabolites in the metabolic networks of taxa that receive the most negative entries on variable 1. The *Enrich. Score* and *FDR-Adj. P* columns show the normalized 'Enrichment score' and FDR-adjusted [8] P-value from the enrichment analysis [5]. The *Synthesized* column indicates whether the network is predicted to produce the metabolite, based on its in-degree [9].

Metabolite	Synthesized	Enrich. Score	FDR-Adj. P
Acyl-glycerophosphoethanolamine	Yes	−2.233	0.00068
Phosphatidylethanolamine	Yes	−2.233	0.00068
L-Lyxose	No	−2.232	0.00068
L-Xylulose	Yes	−2.232	0.00068
CDP diacylglycerol	Yes	−2.230	0.00076

Table 2: Top 5 over-represented metabolites in the metabolic networks of taxa that receive the most negative entries on variable 2. The column descriptions are provided with Supplementary Table 1.

Metabolite	Synthesized	Enrich. Score	FDR-Adj. P
Decaprenyl diphosphate	Yes	2.300	0.00063
Acetyl-cystine-bimane	Yes	2.191	0.00063
Bimane	No	2.191	0.00063
Bimane conjugated mycothiol	Yes	2.191	0.00063
Cys-1D-myo-inositol 2-deoxy-D-glucopyranoside	Yes	2.191	0.00063

Table 3: Top 5 over-represented metabolites in the metabolic networks of taxa that receive the largest positive entries on variable 2. The column descriptions are provided with Supplementary Table 1.

Metabolite	Synthesized	Enrich. Score	FDR-Adj. P
Corrinoid Iron sulfur protein	Yes	3.093	0.026
Methylcorrinoid iron sulfur protein	Yes	3.093	0.026
Citrate	No	2.575	0.038
L-Cystathionine	No	2.468	0.024
Indole	No	2.459	0.011

Table 4: Top 5 over-represented metabolites in the metabolic networks of taxa that receive the largest positive entries on variable 3. The column descriptions are provided with Supplementary Table 1.

Metabolite	Synthesized	Enrich. Score	FDR-Adj. P
Delta(1)-Piperideine-2-carboxylate	Yes	−3.788	0.0003
2 Oxoadipate $C_6H_6O_5$	Yes	−3.788	0.0003
L 2 Aminoadipate $C_6H_10NO_4$	Yes	−3.788	0.0003
L 2 Aminoadipate 6 semialdehyde	Yes	−3.788	0.0003
L-pipecolic acid	Yes	−3.788	0.0003

Table 5: Top 5 over-represented metabolites in the metabolic networks of taxa that receive the most negative entries on variable 4. The column descriptions are provided with Supplementary Table 1.

Metabolite	Synthesized	Enrich. Score	FDR-Adj. P
Riboflavin $C_{17}H_{20}N_4O_6$	No	−2.137	0.000106
L-Histidine	No	−2.124	0.000106
L-Arginine	No	−2.043	0.000107
L-Isoleucine	No	−2.026	0.00012
L-Valine	No	−2.017	0.00012

Table 6: Top 5 over-represented metabolites in the metabolic networks of taxa that receive the most negative entries on variable 8. The column descriptions are provided with Supplementary Table 1.

Metabolite	Synthesized	Enrich. Score	FDR-Adj. P
L-Histidine	No	−1.791	0.002
Tetradecenoyl-CoA	No	−1.782	0.002
Hexadecenoyl-CoA	No	−1.766	0.002
L-Arginine	No	−1.765	0.002
L-Threonine	No	−1.702	0.002

Table 7: Top 5 over-represented metabolites in the metabolic networks of taxa that receive the most negative entries on variable 10. The column descriptions are provided with Supplementary Table 1.
