# Supplementary material for: Mapping the bacterial metabolic niche space
Source: Nat Commun. 2020 Sep 28;11:4887. doi: 10.1038/s41467-020-18695-z (PMC7522980; doi:10.1038/s41467-020-18695-z)
Supplement: Supplementary file 2 — Reporting Summary [file 41467_2020_18695_MOESM2_ESM.pdf]

nature research

Corresponding author(s): Ashkaan K. Fahimipour
Last updated by author(s): 2020/08/28

Reporting Summary

Nature Research wishes to improve the reproducibility of the work that we publish. This form provides structure for consistency and transparency in reporting. For further information on Nature Research policies, see our Editorial Policies and the Editorial Policy Checklist.

Statistics

For all statistical analyses, confirm that the following items are present in the figure legend, table legend, main text, or Methods section.

n/a	Confirmed	
☐	☒	The exact sample size (n) for each experimental group/condition, given as a discrete number and unit of measurement
☒	☐	A statement on whether measurements were taken from distinct samples or whether the same sample was measured repeatedly
☐	☒	The statistical test(s) used AND whether they are one- or two-sided<br>*Only common tests should be described solely by name; describe more complex techniques in the Methods section.*
☐	☒	A description of all covariates tested
☐	☒	A description of any assumptions or corrections, such as tests of normality and adjustment for multiple comparisons
☐	☒	A full description of the statistical parameters including central tendency (e.g. means) or other basic estimates (e.g. regression coefficient) AND variation (e.g. standard deviation) or associated estimates of uncertainty (e.g. confidence intervals)
☐	☒	For null hypothesis testing, the test statistic (e.g. F, t, r) with confidence intervals, effect sizes, degrees of freedom and P value noted<br>*Give P values as exact values whenever suitable.*
☒	☐	For Bayesian analysis, information on the choice of priors and Markov chain Monte Carlo settings
☒	☐	For hierarchical and complex designs, identification of the appropriate level for tests and full reporting of outcomes
☐	☒	Estimates of effect sizes (e.g. Cohen's d, Pearson's r), indicating how they were calculated

Our web collection on statistics for biologists contains articles on many of the points above.

Software and code

Policy information about availability of computer code

Data collection	Publicly available data were accessed from NCBI as described in the Methods section of the main text, using the ncbi-genome-download software: https://github.com/kblin/ncbi-genome-download
Data analysis	Custom software has been made available on a public Figshare repository. Data were analyzed using the open source R programming language (4.0.0). Specific libraries are referenced in the Methods section of the main text. Other software used for analyses include: GToTree 1.4.8, FastTree 2.1.10, BLAST 2.9.0+, and the CarveMe software (https://github.com/cdanielmachado/carveme).

For manuscripts utilizing custom algorithms or software that are central to the research but not yet described in published literature, software must be made available to editors and reviewers. We strongly encourage code deposition in a community repository (e.g. GitHub). See the Nature Research guidelines for submitting code & software for further information.

Data

Policy information about availability of data

All manuscripts must include a data availability statement. This statement should provide the following information, where applicable:

- Accession codes, unique identifiers, or web links for publicly available datasets
- A list of figures that have associated raw data
- A description of any restrictions on data availability

NCBI RefSeq genome accession numbers are available at the Figshare repository: https://doi.org/10.6084/m9.figshare.12864011.v4. The Earth Microbiome data base was accesed via ftp: ftp://ftp.microbio.me/emp/release1

Field-specific reporting

Please select the one below that is the best fit for your research. If you are not sure, read the appropriate sections before making your selection.

☐ Life sciences ☐ Behavioural & social sciences ☒ Ecological, evolutionary & environmental sciences

For a reference copy of the document with all sections, see nature.com/documents/nr-reporting-summary-flat.pdf

Ecological, evolutionary & environmental sciences study design

All studies must disclose on these points even when the disclosure is negative.

Study description	In this study, we present a manifold learning approach to organize and systematize the wealth of publicly available bacterial genomes published in recent years. Bacterial genomic and proteomic data were accessed directly from the National Center for Biotechnology Information RefSeq database. We analyzed a total of 2,621 bacterial genomes, which included a single randomly-selected representative from all unique genera in the database.
Research sample	No new data were collected for the purpose of this study. The analyzed genome-scale metabolic networks represent a large fraction of known biochemical capabilities for many if not most currently known bacterial genera. These data were accessed directly from the RefSeq database, as well as from the public Earth Microbiome Project FTP server (ftp://ftp.microbio.me/emp/release1/otu_tables/).
Sampling strategy	Subsampling of all available RefSeq genomes is described in the main text, and included one representative from all genera with assemblies in the database.
Data collection	No new data were collected for this study.
Timing and spatial scale	No new data were collected for this study. The RefSeq database was accessed on March 20, 2019.
Data exclusions	No data were excluded from analyses.
Reproducibility	No experiments were performed. Our analyses can be reproduced using the demo scripts provided in the statistical programming language, R (https://github.com/AshkaanF/diffusion_maps).
Randomization	This is not relevant to our study, as we present a new way to analyze large amounts of publicly available data.
Blinding	Blinding is not relevant to our study, as no trials were performed.

Did the study involve field work? ☐ Yes ☒ No

Reporting for specific materials, systems and methods

We require information from authors about some types of materials, experimental systems and methods used in many studies. Here, indicate whether each material, system or method listed is relevant to your study. If you are not sure if a list item applies to your research, read the appropriate section before selecting a response.

Materials & experimental systems

n/a	Involved in the study
☒	☐ Antibodies
☒	☐ Eukaryotic cell lines
☒	☐ Palaeontology and archaeology
☒	☐ Animals and other organisms
☒	☐ Human research participants
☒	☐ Clinical data
☒	☐ Dual use research of concern

Methods

n/a	Involved in the study
☒	☐ ChIP-seq
☒	☐ Flow cytometry
☒	☐ MRI-based neuroimaging
